# Metabonomics analysis of flavonoids in seeds and sprouts of two Chinese soybean cultivars

**DOI:** 10.1038/s41598-022-09408-1

**Published:** 2022-04-01

**Authors:** Weiwei Bi, Guixing Zhao, Yutong Zhou, Xiaoyu Xia, Jinsheng Wang, Guangjin Wang, Shuwen Lu, Wenjin He, Taifei Bi, Jinrong Li

**Affiliations:** 1 Heilongjiang Academy of Agricultural Sciences Post-Doctoral Station, Nangang District, Harbin, 150086 People’s Republic of China; 2 Soybean Research Institute, Heilongjiang Academy of Agricultural Sciences, Nangang District, Harbin, 150086 People’s Republic of China; 3 Colleage of Life Sciences, Yulin University, Yuyang District, Yulin, 719000 People’s Republic of China; 4 School of Hydraulic and Electric Power, Heilongjiang University, Nangang District, Harbin, 150086 People’s Republic of China; 5 Food Processing Research Institute, Heilongjiang Academy of Agricultural Sciences, Nangang District, Harbin, 150086 People’s Republic of China

**Keywords:** Biochemistry, Plant sciences

## Abstract

A popular food in China, soybean seeds and sprouts contained many biologically active substances which are beneficial to the human body, such as flavonoids. Northeast of China is the main producing area of soybean. The experimental materials came from the main soybean producing areas in Northeast China, this study compared flavonoids of two China cultivars of soybeans, Heinong52(HN52) and Heinong71(HN71). Here, we also considered the effects of germination on the chemical profile of flavonoids. Using a LC–ESI–MS/MS system, 114 differential flavonoid metabolites were identified. A total of 18 metabolites were significantly different between the two soybean varieties before germination, of which 14 were up-regulated and 4 were down-regulated. After germination, 33 significantly different metabolites were found in the two soybean sprouts, of which 19 were up-regulated and 14 were down-regulated. These experimental results revealed significant up-regulation of metabolites in soybean sprouts compared with soybean seeds, thus suggesting that soybean germination may increase content of flavonoid metabolites. There are six main pathways for the synthesis of flavonoids: isoflavonoid biosynthesis, flavonoid biosynthesis, flavone and flavonol biosynthesis, biosynthesis of secondary metabolites, and biosynthesis of phenylpropanoids. Soybean seeds lack flavone and flavanol biosynthesis and develop the capacity for this biosynthetic pathway after germination as sprouts. Isoflavonoid biosynthesis is the most abundantly utilized pathway.

## Introduction

China is the first country to grow and domesticate the soybean(*Glycine max*(L.) Merr.), with a cultivation history of at least 4,000 years. Nowadays, soybean is gaining popularity in many countries largely as a vegetable protein and oil source, owing to its bean composition of approximately 40% protein and 20% oil^1^. Soybean is an important nutritional component of diets and is used in many foods, such as soybean oil, soybean sprout, paste, soymilk, and tofu^2^. Mostly present in plant leaves and fruits, flavonoids primarily exist as glycosides combining with sugars. An important phenolic secondary metabolite in plants, flavonoids can support plant disease resistance, and in the human body have roles such as anti-oxidation, anti-cancer, and anti-aging^3,4^. Certain major flavonoids, such as flavonols, flavonol glycosides, isoflavones, chalcones, anthocyanins and procyanidins have been previously isolated and identified from soybeans^5,6^. Additionally, recent research showed that pinto and black beans as excellent dietary sources of natural antioxidants and thus may serve as disease preventative and healthy foods^7^. Moreover, a recent review analyzing epidemiological studies in the last ten years and the flavonoid content of diets concluded that dietary flavonoids may prevent a range of different types of cancer^8^. In fact, free phenolic compounds, such as flavonols and isoflavones, have been found to have with immunomodulatory and cellular antiproliferative functions^9^.

The sprouting process induces a variety of biochemical changes in soybean seeds, leading to the accumulation of primary and secondary metabolites^10^. Some studies have found that germination and sprout formation of legumes are accompanied by a significant increase in total flavonoids content with antioxidant potential^11–13^. Generally, content of genistein derivatives is about two times higher than that of daidzein derivatives in raw soybeans^14–16^. Germination can induce isoflavone profile changes in soybeans including decreases of β-glycosides and increases of both 6ʹ-O-malonyl-β-glucosides and aglycones^17–19^. During germination, a 13% increase of total isoflavone content was found by Kim, et al^20^. One comparative study of the chemical profiles of three Egyptian cultivars of fava beans; Sakha 3, Nubaria 3, and Giza 843, focused on the effects of germination on the chemical profiles of phenolic compounds and saponins and characterized 65 metabolites based on UV spectra, accurate MS, and MS/MS data. Germination was found to dramatically increase the quantities of flavonoids and saponins to which biological activities are attributed^20^. A previous study has suggested that germination could enhance the content of isoflavonesin soybeans^21^. Accordingly, studies based on metabolomics have applied combined techniques, such as chromatographic separation (UHPLC and/or gas chromatography) and mass spectrometry, for comprehensive metabolic analysis of plant substrates^22,23^. Recent technological developments in the field of metabolomics, especially as a result of the widespread use of LC-high resolution MS have considerably improved accuracy and sensitivity of metabolite detection^24^. For example, non-target LC–MS-Orbitrap metabolomics methods were used to compare and analyze the biologically active metabolites of frozen, boiled, and canned chickpeas, lentils, and white beans^25^. The main purposes of this study were to: (1) compare the flavonoid contents in soybean seeds and sprouts grown under dark conditions for 3 days at 24 °C (2) analyze the metabolism of flavonoids in soybean sprouts.

## Materials and methods

### Samples preparation

According to our previous detection of isoflavones in 12 main soybean varieties grown in Heilongjiang, China, two varieties with high isoflavone content were selected for experimentation^26^. The two soybean cultivars Heinong52 (HN52), and Heinong71(HN71) used in this experiment were cultivated at the Harbin Soybean Research Institute (Modern Agriculture Demonstration Zone of Heilongjiang Academy of Agricultural Sciences, Minzhu Township, Daowai District, Harbin; 126.65°E, 45.78°N), Heilongjiang Academy of Agricultural Sciences, Harbin, China(statement as follow). Soybean seeds were harvested from three replicates of each cultivar for a cropping year and stored at room temperature until analysis of flavonoids compounds. Briefly, high-quality soybean grains were selected, rinsed with clean water, soaked in distilled water at 24 °C for 4 h, spread in a single layer on a tray, covered with gauze, and placed in an artificial climate box (HPG-1600H, Harbin Donglian Electronic Technology Development Co., Ltd. Harbin, China) at about 24 °C and 80% humidity to germinate for 3 days in the dark. The growth chamber was set up to spray water for 3 min every 1 h. After germination, approximately 20 g FW(fresh weight) of soybean sprouts (HNS52,HNS71) per cultivar with three replicates every sampling day were randomly collected from containers, and freeze-dried at − 50 °C for 20 h. The freeze-dried sprouts and seeds were crushed using a mixer mill (MM 400, Retsch) with a zirconia bead for 1.5 min at 30 Hz. 100 mg powder was weighed and extracted overnight at 4 °C with 1.0 mL 70% aqueous methanol. Following centrifugation at 10,000 g for 10 min, the extracts were absorbed (CNWBOND Carbon-GCB SPE Cartridge, 250 mg, 3 mL; ANPEL, Shanghai, China, www.anpel.com.cn/cnw) and filtrated (SCAA-104,0.22 μm pore size; ANPEL, Shanghai, China, http://www.anpel.com.cn/) LC–MS analysis(contained “2.2 HPLC Conditions” and “2.3 ESI-Q TRAP-MS/MS”) .

### HPLC conditions

The sample extracts were analyzed using a LC–ESI–MS/MS system (HPLC, Shim-pack UFLC SHIMADZU CBM30A system, www.shimadzu.com.cn/; MS, Applied Biosystems 4500 Q TRAP, www.appliedbiosystems.com.cn/). We used the following steps to analyze, HPLC: column, Waters ACQUITY UPLC HSS T3 C18 (1.8 µm, 2.1 mm*100 mm); solvent system, water (0.04% acetic acid): acetonitrile (0.04% acetic acid); gradient program,100:0 V/V at 0 min, 5:95 V/V at 11.0 min, 5:95 V/V at 12.0 min, 95:5 V/V at 12.1 min, 95:5 V/V at 15.0 min; flow rate, 0.40 mL/min; temperature, 40 °C; injection volume: 5μL. The effluent was alternatively connected to an ESI-triple quadrupole-linear ion trap (QTRAP)-MS^27^.

LIT and triple quadrupole (QQQ) scans were acquired using a triple quadrupole-linear ion trap mass spectrometer (Q TRAP), API 4500 Q TRAP LC/MS/MS System, equipped with an ESI Turbo Ion-Spray interface, operating in a positive ion mode and controlled by Analyst 1.6.3 software (AB Sciex). The parameters of ESI source operation were as follows: ion source, turbo spray; source temperature 550 °C; ion spray voltage (IS)5500 V; ion source gas I (GSI), gas II(GSII), curtain gas (CUR) were set at 55, 60, and 25.0 psi, respectively; the collision gas(CAD) was high. Instrument tuning and mass calibration were performed with 10 and 100 μmol/L polypropylene glycol solutions in QQQ and LIT modes, respectively. QQQ scans were acquired as MRM experiments with collision gas (nitrogen) set to 5 psi. DP and CE for individual MRM transitions was done with further DP and CE optimization. A specific set of MRM transitions were monitored for each period according to the metabolites eluted within this period^28–30^. Similarly, Valente et al. calculated the metabolic profile and antioxidant activity of 7 European broad bean seed varieties^31^ and their pods (by-products) using HPLC–DAD-MS/MS-based methods and found the existence of 105 phenolic compounds, alkaloids, jasmonic acids, and organic acids^32^.

### Statistics analysis

For all experiments, three independent assays were carried out. One-way analysis of variance was used to study the differences between means, with a significant level at *P* < 0.05. Data analysis was carried out with SPSS (Windows version 12.0, SPSS Inc., Chicago,IL, USA). All data are presented as mean ± standard error of means.

## Results and discussion

### Principal component analysis

**Figure 1:**
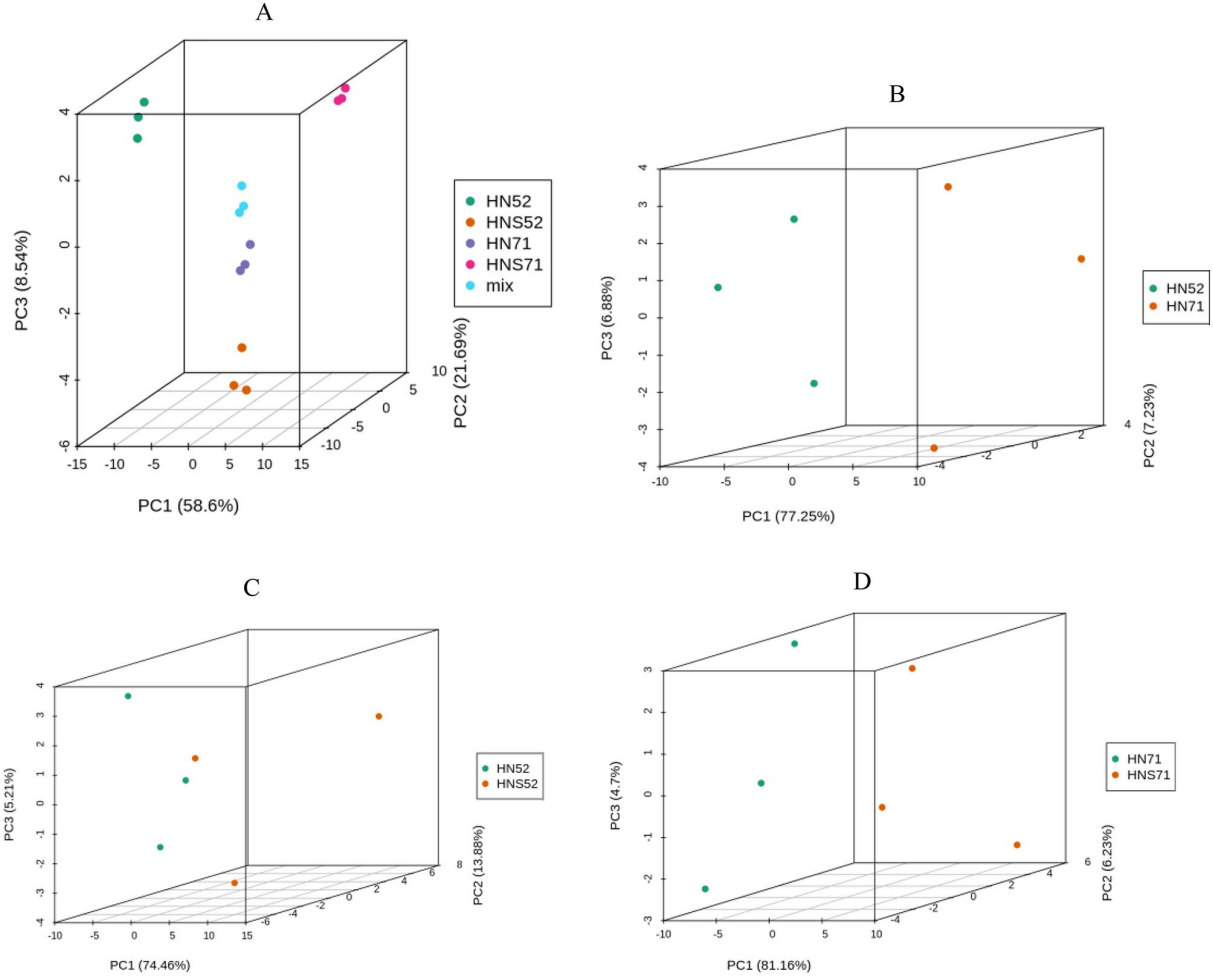
PCA score chart of mass spectrum data of each group of samples and quality control samples. Mix: quality control samples, (**A**): all samples of PCA. (**B**): Two soybean seeds of PCA; (**C**):Soybean and sprouts of PCA(HN52 and HNS52); (**D**): Soybean and sprouts of PCA(HN71 and HNS71).

Principal component analysis (PCA) was performed on metabolic data to visualize the biological variability of soybean seeds and sprouts. These results indicated that the dominating source of variance was the differential flavonoid content in the two soybean varieties across all germination stages (PC1). From Fig. 1A, the PC1 of 4 groups and mix was 58.6%. The PC1 of two soybean seeds was 77.25%. The PC1 of two soybean seeds and sprouts were74.46% and 81.16% respectively. The PC1 of all samples was greater than 50%. These results indicated that the grouping of samples is reasonable. Through PCA of samples (including quality control samples MIX), in order to have a preliminary understanding of the overall metabolic differences between samples in each group and the degree of variability between samples within the group. The PCA results show the trend of metabolome separation between groups, suggesting whether there is a difference in metabolome between sample groups. The X axis represents the first principal component, and the Y axis represents the second principal component. Grouping principal component analysis: before doing difference analysis, first perform principal component analysis on the grouped samples for difference comparison to observe the degree of variability between the difference groups and the samples within the group.

### Differential metabolic analysis of flavonoids

From Table 1, a total of 114 flavonoid metabolites were detected in the two soybean varieties and their sprouts. These consisted of 41 isoflavones, 26 flavonoids, 14 flavonols, 10chalcones, 6 dihydroflavones, 5 tannins, 4 dihydroflavonols, 3 carbonosides, 2 flavonoids, 1 flavanol, 1 proanthocyanidin, and 1anthocyanin. These results indicate that the flavonoids in soybeans and sprouts are mainly composed of isoflavones (35.96%). 13 substances detected in this study were the same as those detected in Egyptian cultivars of chickpea in a previous study^30^. These were C_13_H_16_O_10_ (6-O-Galloyl-glucose), C_13_H_16_O_10_(3-O-Galloyl-glucose),C_13_H_16_O_10_ (2-O-Galloyl-glucose), C_27_H_30_O_16_(Kaempferol-3-O-neohesperidoside), C_27_H_30_O_16_ (5,7-Dihydroxy-4-methoxyflavone-3-O-xylose-(1–6)-glucose), C_21_H_22_O_11_ (Aromadendrin-7-O-glucoside), C_27_H_30_O_16_ (Quercetin-3-O-robinobioside), C_27_H_30_O_17_ (Quercetin-3-O-sophoroside (Baimaside)), C_33_H_40_O_21_ (Kaempferol-3-O-sophoroside -7-O- glucoside) , C_27_H_32_O_15_ (Eriodictyol-7-O-Rutinoside (Eriocitrin)), C_27_H_30_O_16_ (Quercetin-3-O-rutinoside (Rutin)), C_28_H_32_O_17_ (2'-Hydoxy, 5-methoxy Genistein-4',7- O-diglucoside), C_27_H_30_O_15_ (Eriodictyol-7-O-Rutinoside (Eriocitrin)), respectively. This indicated that these flavonoids may be universal in beans.

**Table 1:** Flavonoids in soybeans seeds and sprouts.

Index	Formula	Compounds	Class	CAS
pme0309	C_8_H_8_O_5_	3-O-Methylgallic Acid	Tannin	3934-84-7
pme0355	C_15_H_10_O_4_	Daidzein	Isoflavones	486-66-8
mws0902	C_15_H_12_O_4_	Liquiritigenin	Dihydroflavone	578-86-9
pme3217	C_15_H_12_O_4_	Isoliquiritigenin	Chalcones	961-29-5
mws0037	C_16_H_12_O_4_	Formononetin (7-Hydroxy-4'-methoxyisoflavone)	Isoflavones	485-72-3
mws0063	C_15_H_10_O_5_	Genistein*	Isoflavones	446-72-0
pme3261	C_15_H_10_O_5_	6-Hydroxydaidzein*	Isoflavones	17,817-31-1
mws0912	C_15_H_10_O_5_	2'-Hydroxydaidzein*	Isoflavones	7678-85-5
pmp000344	C_15_H_10_O_5_	3',4',7-Trihydroxyflavone*	Flavonoid	2150-11-0
Lmmp005125	C_16_H_14_O_4_	Pinostrobin Chalcone	Chalcones	18956-15-5
mws4060	C_16_H_14_O_4_	Echinatin	Chalcones	34221-41-5
pme1397	C_15_H_11_O_5_ +	Pelargonidin	Anthocyanins	7690-51-9
mws0914	C_15_H_12_O_5_	Pinobanksin*	Dihydroflavonol	548-82-3
pme0376	C_15_H_12_O_5_	Naringenin (5,7,4'-Trihydroxyflavanone)*	Dihydroflavone	480-41-1
pme3250	C_16_H_12_O_5_	Biochanin A	Isoflavones	491-80-5
pme3233	C_16_H_12_O_5_	Calycosin*	Isoflavones	20575-57-9
mws0908	C_16_H_12_O_5_	Glycitein*	Isoflavones	40957-83-3
mws0062	C_15_H_10_O_6_	Isoluteolin (Orobol)(5,7,3',4'-tetrahydroxyisoflavone)	Isoflavones	480-23-9
pme0088	C_15_H_10_O_6_	Luteolin (5,7,3',4'-Tetrahydroxyflavone)	Flavonoid	491-70-3
mws1094	C_15_H_12_O_6_	Dihydrokaempferol	Dihydroflavonol	480-20-6
Lmdp005525	C_17_H_14_O_5_	Afrormosin	Isoflavones	550-79-8
Lmqn003210	C_17_H_14_O_5_	5-Hydroxy-6,7-dimethoxyflavone	Flavonoid	740-33-0
Zmhp003514	C_16_H_12_O_6_	6,7,8-Tetrahydroxy-5-methoxyflavone*	Flavonoid	–
mws0058	C_16_H_12_O_6_	Diosmetin (5,7,3'-Trihydroxy-4'-methoxyflavone)	Flavonoid	520-34-3
pmp000001	C_16_H_12_O_6_	Hispidulin (5,7,4'-Trihydroxy-6-methoxyflavone)*	Flavonoid	1447-88-7
pmp000804	C_19_H_20_O_4_	Isobavachalcone D	Chalcones	–
pmp000348	C_20_H_18_O_4_	Kanzonol D	Other Flavonoids	155,233-20-8
Lmfn000604	C_13_H_16_O_10_	6-O-Galloyl-glucose*	Tannin	13,186-19-1
Hmln000659	C_13_H_16_O_10_	3-O-Galloyl-glucose*	Tannin	–
Hmln000873	C_13_H_16_O_10_	2-O-Galloyl-glucose	Tannin	–
pmp000350	C_20_H_16_O_5_	Glabrone	Isoflavones	60008-02-8
pmp001223	C_20_H_18_O_5_	Psoralenol	Isoflavones	70522-30-4
pmp000352	C_20_H_18_O_5_	Licoflavone C	Flavonoid	72357-31-4
pmp000351	C_20_H_18_O_5_	Eurycarpin A	Isoflavones	166547-20-2
pmp000637	C_20_H_20_O_5_	Sophoraflavanone B	Dihydroflavone	53846-50-7
Xmgp006913	C_20_H_20_O_5_	2,4,2',4'-tetrahydroxy-3'-prenylchalcone	Chalcones	–
pmn001389	C_21_H_20_O_5_	Gancaonin G	Isoflavones	126716-34-5
pmp000355	C_20_H_18_O_6_	Licoflavonol	Flavonols	60197-60-6
pmp000811	C_20_H_18_O_6_	Morachalcone C	Chalcones	1304549-24-3
pmp000360	C_21_H_22_O_5_	3-Hydroxylicochalcone A	Chalcones	–
pmp000361	C_21_H_22_O_5_	Licochalcone D	Chalcones	144506-15-0
pmp001224	C_20_H_20_O_6_	Brosimacutin G	Chalcones	350,221-50-0
Cmsn000894	C_14_H_18_O_11_	7-O-Galloyl-D-sedoheptulose	Tannin	–
mws0055	C_20_H_20_O_7_	Tangeretin	Flavonols	481-53-8
pmp000417	C_21_H_20_O_9_	Daidzein-4'-O-glucoside	Isoflavones	–
pme1587	C_21_H_20_O_9_	Daidzein-7-O-glucoside(Daidzin)	Isoflavones	552-66-9
mws1597	C_21_H_20_O_9_	Puerarin	Isoflavones	3681-99-0
pmp000384	C_21_H_22_O_9_	Isoliquiritin*	Chalcones	5041-81-6
pmp000383	C_21_H_22_O_9_	Liquiritigenin-4'-O-Glucoside (Liquiritin)*	Dihydroflavone	551-15-5
pmp000647	C_25_H_28_O_6_	Kushenol E	Other Flavonoids	99,119-72-9
pme3504	C_22_H_22_O_9_	Formononetin-7-O-glycoside (Ononin)	Isoflavones	486-62-4
HJN089	C_21_H_20_O_10_	Sophoricoside	Isoflavones	152-95-4
pmp000413	C_21_H_20_O_10_	Genistein-8-C-glucoside*	Isoflavones	66,026-80-0
mws1434	C_21_H_20_O_10_	Apigenin-6-C-glucoside (Isovitexin)*	Flavonoid carbonoside	29,702-25-8
Lmlp005572	C_21_H_20_O_10_	Galangin-7-O-glucoside*	Flavonoid	–
mws0072	C_21_H_20_O_10_	Apigenin-5-O-glucoside*	Flavonoid	28,757-27-9
mws0895	C_21_H_20_O_10_	Genistein-7-O-Glucoside (Genistin)*	Isoflavones	529-59-9
pme1611	C_21_H_22_O_10_	Isohemiphloin*	Flavonoid carbonoside	3682-02-8
mws1179	C_21_H_22_O_10_	Naringenin-7-O-glucoside (Prunin)*	Dihydroflavone	529-55-5
HJN090	C_21_H_22_O_10_	Butin-7-O-glucoside*	Flavonoid	–
pmp000550	C_22_H_22_O_10_	Calycosin-7-O-glucoside	Isoflavones	20,633-67-4
HJN091	C_22_H_20_O_10_	Prunetin-4'-O-glucoside	Isoflavones	154-36-9
pme3400	C_22_H_22_O_10_	Biochanin A-7-O-glucoside (Sissotrin)	Isoflavones	5928-26-7
pmp000415	C_22_H_22_O_10_	3'-Methoxydaidzin	Isoflavones	200,127-80-6
mws0894	C_22_H_22_O_10_	Glycitin	Isoflavones	40,246-10-4
mws1172	C_22_H_22_O_10_	Trifolirhizin (Maackiain-3-O-glucoside)	Isoflavones	6807-83-6
Lmlp003531	C_21_H_20_O_11_	Luteolin-3'-O-glucoside*	Flavonoid	5154-41-6
pme2459	C_21_H_20_O_11_	Luteolin-7-O-glucoside (Cynaroside)*	Flavonoid	5373-11-5
Xmyp005654	C_21_H_20_O_11_	Kaempferol-4'-O-glucoside*	Flavonoid	–
mws1361	C_21_H_22_O_11_	Astilbin	Dihydroflavonol	29838-67-3
Lmtn002796	C_21_H_22_O_11_	Aromadendrin-7-O-glucoside	Flavonoid	28189-90-4
Lmlp005236	C_21_H_22_O_11_	Dihydrokaempferol-3-O-glucoside	Dihydroflavonol	1049-08-8
Zmdp004370	C_23_H_22_O_10_	6''-O-Acetyldaidzin	Isoflavones	71385-83-6
Lmdp003994	C_23_H_24_O_10_	6,4'-Dimethoxyisoflavone-7-O-glucoside (Wistin)	Isoflavones	19046-26-5
mws0091	C_21_H_20_O_12_	Quercetin-3-O-glucoside (Isoquercitrin)	Flavonols	482-35-9
mws0061	C_21_H_20_O_12_	Quercetin-3-O-galactoside (Hyperin)	Flavonols	482-36-0
pmp000191	C_23_H_22_O_11_	6''-O-Acetylgenistin*	Isoflavones	73566-30-0
pmp000581	C_23_H_22_O_11_	Apigenin-7-O-(6''-acetyl)glucoside*	Flavonoid	72741-92-5
pmp000192	C_24_H_24_O_11_	Acetylglycitin	Isoflavones	134859-96-4
Lmmp003903	C_23_H_22_O_12_	Kaempferol-3-O-(2''-acetyl)glucoside	Flavonols	–
Lmmn003398	C_23_H_22_O_12_	Kaempferol-3-O-(6''-acetyl)glucoside	Flavonols	–
Lmgp004829	C_23_H_24_O_12_	5,7,4'-Trihydroxy-6,8-dimethoxyisoflavone-7-O-galactosides	Isoflavones	–
pmp000193	C_24_H_22_O_12_	6''-O-Malonyldaidzin	Isoflavones	124590-31-4
Zmdp004305	C28H24O9	Daidzein-7-O-(2''-benzoyl)rhamnoside	Flavonoid	-
Zmdp005767	C_25_H_24_O_12_	Formononetin-7-O-(6''-Malonyl)glucoside	Isoflavones	-
Zmdp004112	C_24_H_22_O_13_	Genistein-7-O-(6''-malonyl)glucoside	Isoflavones	-
pmp000194	C_24_H_22_O_13_	6''-O-Malonylgenistin	Isoflavones	51011-05-3
pmp000195	C_25_H_24_O_13_	6''-O-Malonylglycitin	Isoflavones	137705-39-6
Lmmp003817	C_24_H_22_O_14_	Kaempferol-3-O-(6''-malonyl)glucoside*	Flavonols	–
pmp000587	C_24_H_22_O_14_	Luteolin-7-O-(6''-malonyl)glucoside*	Flavonoid	–
Lmdp004892	C_24_H_22_O_14_	Kaempferol-3-O-(6''-malonyl)galactoside*	Flavonols	–
pmb0608	C_25_H_24_O_14_	Chrysoeriol-7-O-(6''-malonyl)glucoside	Flavonoid	–
Zmdp003677	C_26_H_28_O_13_	Daidzein-7-O-Glucoside-4'-O-Apioside	Flavonoid	108069-01-8
Wmkn002777	C_27_H_30_O_13_	7-Hydroxy-3''-methoxy-isoflavone-7-primeveroside	Isoflavones	–
Lmnp202580	C_26_H_28_O_14_	Apigenin-8-C-(2''-xylosyl)glucoside	Flavonoid carbonoside	–
pmp000411	C_26_H_28_O_14_	Apigenin-7-O-(2''-glucosyl)arabinoside	Flavonoid	–
mws0836	C_30_H_26_O_12_	Procyanidin B1	Proanthocyanidins	20315-25-7
pmp000414	C_27_H_30_O_14_	Puerarin-4'-O-glucoside	Isoflavones	117047-08-2
pmp001079	C_27_H_30_O_15_	Luteolin-7-O-neohesperidoside (Lonicerin)	Flavonoid	25694-72-8
mws1073	C_27_H_30_O_15_	Apigenin-6,8-di-C-glucoside	Flavonoid	23666-13-9
Lmjp002867	C_27_H_30_O_15_	Kaempferol-3-O-neohesperidoside	Flavonols	32602-81-6
Wmkp002590	C_27_H_30_O_15_	5,7-Dihydroxy-4-methoxyflavone-3-O-xylose-(1-6)-glucose	Flavonoid	–
mws1519	C_27_H_32_O_15_	Eriodictyol-7-O-Rutinoside (Eriocitrin)	Dihydroflavone	13463-28-0
Lmmp000897	C_30_H_26_O_14_	Gallocatechin-Gallocatechin	Flavanols	–
pmn001583	C_27_H_30_O_16_	Quercetin-3-O-robinobioside*	Flavonols	52525-35-6
Zmxp003107	C27H30O16	Luteolin-7,3'-di-O-glucoside*	Flavonoid	52187-80-1
mws0059	C_27_H_30_O_16_	Quercetin-3-O-rutinoside (Rutin)*	Flavonols	153-18-4
Lmhp003217	C_28_H_32_O_16_	2'-Hydoxy,5-methoxyGenistein-O-rhamnosyl-glucoside	Isoflavones	–
pme1540	C_28_H_32_O_16_	Isorhamnetin-3-O-neohesperidoside	Flavonols	55033-90-4
Lmtp003677	C_27_H_30_O_17_	Quercetin-3-O-sophoroside (Baimaside)	Flavonols	18609-17-1
Lmhp002800	C_28_H_32_O_17_	2'-Hydoxy,5-methoxyGenistein-4',7-O-diglucoside	Isoflavones	–
Hmqp003435	C_29_H_30_O_17_	Apigenin-7-O-(2''-O-apiosyl)(6''-Malonyl)glucoside	Flavonoid	–
Hmlp003185	C_30_H_32_O_18_	Luteolin-7-O-(6''-malonyl)glucoside-5-O-rhamnoside	Flavonoid	–
Lmwp003888	C_33_H_40_O_21_	Kaempferol-3-O-sophoroside-7-O-glucoside	Flavonols	–

**Figure 2:**
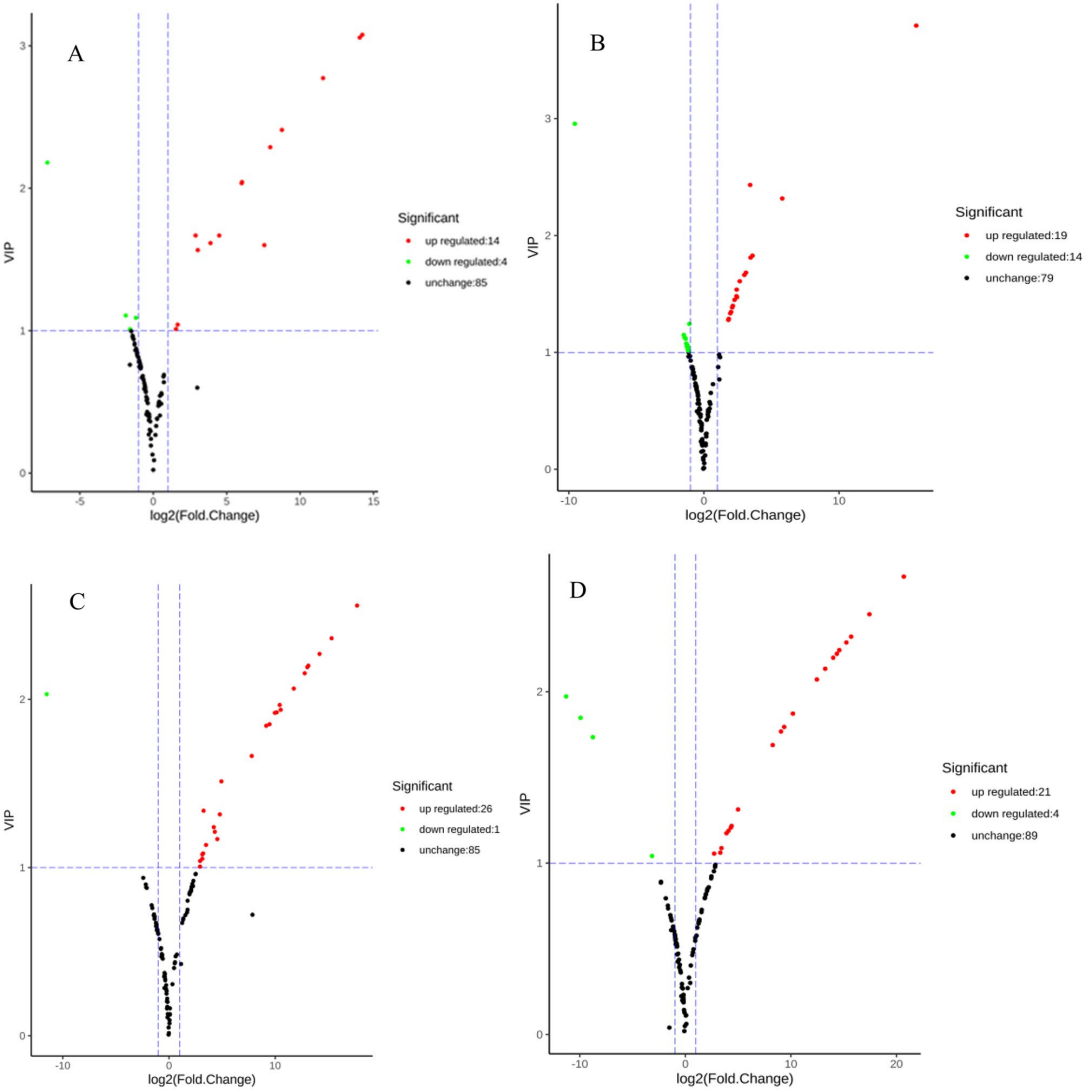
Volcano Map of Differential Metabolites. (**A**): HN52 vs HN71; (**B**): HNS52 vs HNS71; (**C**): HN52 vs HNS52; (**D**): HN71 vs HNS71.

Metabolomic data is massive and multidimensional, so it is imperative to combine univariate and multivariate statistical analyses, to analyze the data from a multivariate perspective according to the characteristics of the data, and to accuratelymine differential metabolites. This study has three biological replicates. The method of combining fold change ≥ 2 and OPLS-DA model VIP (The VIP value represents the influence of the difference between the corresponding metabolites in the classification and discrimination of each group of samples in the model. It is generally believed that the metabolites with VIP ≥ 1 are significantly different.) ≥ 1 was used to screen for differential metabolites. Shown in Fig. 2A, there were a total of 18 significantly different metabolites between the two soybean varieties, of which 14 were up-regulated and 4 were down-regulated. After germination, the significantly different metabolites in the two soybean sprouts were 33, of which 19 were up-regulated and 14 were down-regulated (Fig. 2B). Moreover, there were a total of 27 significantly different metabolites between HN52 and HNS52 cultivars, of which 26 were up-regulated and 1 were down-regulated (Fig. 2C). Additionally, there were a total of 25 significantly different metabolites between HN71 and HNS71, of which 21 were up-regulated and 4 were down-regulated (Fig. 2D). These experimental results showed that both differential and up-regulated metabolites increased in soybean sprouts compared to soybean seeds, thus indicating that soybean germination can increase content of flavonoid metabolites. From a qualitative comparison, that the germination process clearly has an impact on the number of characterized metabolites, especially flavonoid compounds. In fact, the biological activity of the plant substrate is mainly due to these metabolites^33–37^.

Each point in the volcano map represents a metabolite. The abscissa represents the logarithm of the quantitative difference of a certain metabolite in the two samples; the ordinate represents the VIP value. The larger the absolute value of the abscissa, the greater the multiple difference in the expression amount between the two samples; the larger the ordinate value, the more significant the differential expression, and the more reliable the differentially expressed metabolites screened. The green dots in the figure represent down-regulated differentially expressed metabolites, the red dots represent up-regulated differentially expressed metabolites, and black represents detected but not significantly different metabolites.

**Figure 3:**
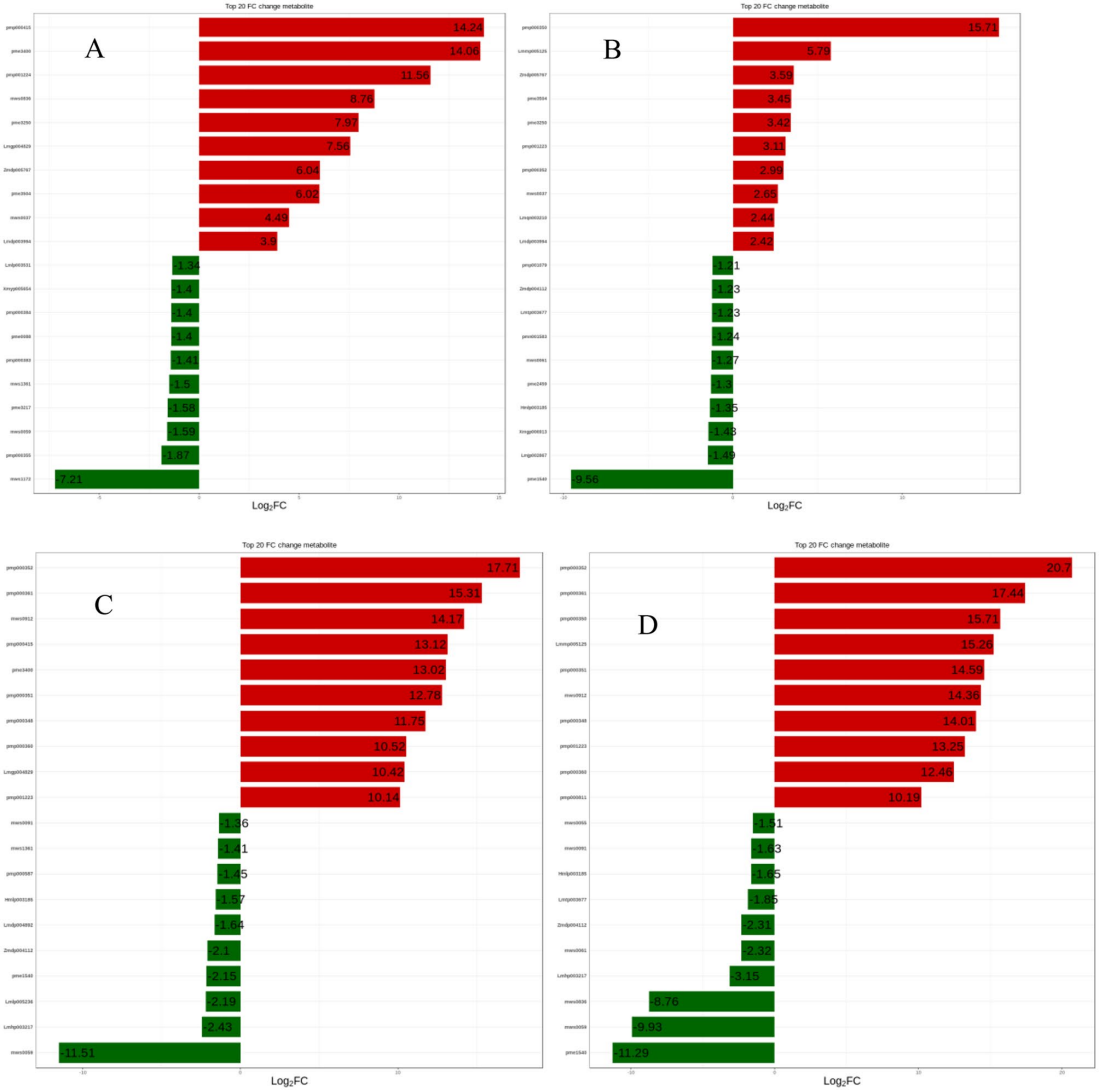
Analysis of the fold difference of metabolites between different samples. (**A**): HN52 vs HN71; (**B**): HNS52 vs HNS71; (**C**): HN52 vs HNS52; (**D**): HN71 vs HNS71.

As shown in Fig. 3, the flavonoids of the two varieties have a balanced distribution of the differential metabolite multiples, and the differential metabolites multiples decrease after germination. This indicated that the flavonoid metabolism of different varieties of soybeans while germinating was highly similar. The flavonoid metabolites in the same variety of soybean seeds and sprouts differ greatly in multiples, showing that flavonoid metabolism is especially active during soybean germination, and that germination is a way to accumulate flavonoids. During the growth process, small sprouts may be damaged by microbial and environmental pressures or nutrient deficiency. Therefore it is necessary for plants to synthesize secondary metabolites (such as flavonoid compounds) through different metabolic pathways to develop defense mechanisms^38–40^.

**Figure 4:**
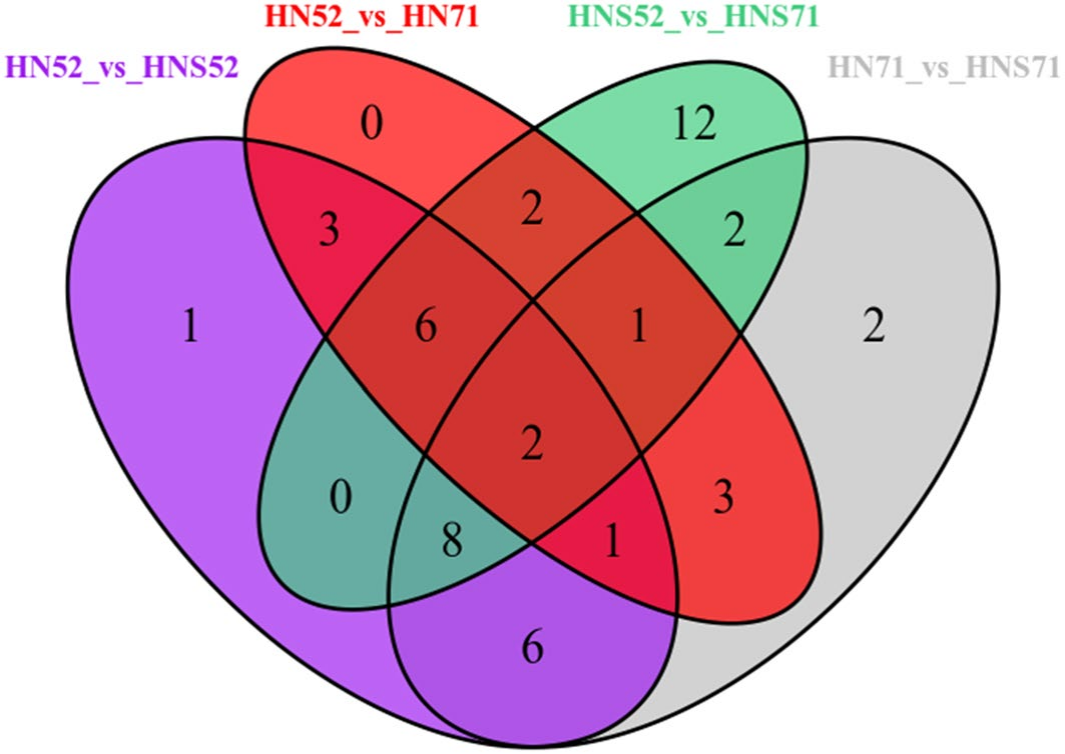
Venn diagram showing the relationships between different groups of metabolites.

As shown in Fig. 4, there were total 33 differential metabolites, of which 12 identical metabolites in HN52 vs HNS52 and HN52 vs HN71. There were total 45 differential metabolites, of which 13 metabolites were shared in HN71 vs HNS71 and HNS52 vs HNS71. These results indicated that flavonoid metabolites increased in germinated soybeans. For example, Wu et al. discussed the effect of germination on the antioxidant potential and isoflavonoid content of chickpeas and observed increases in both antioxidant activity and isoflavonoids content with germination^41^. Additionally, Ayet et al. revealed that germination increased the content of soya saponins in lentils^42,43^. Moreover, Mekky et al. revealed that germination increased the quantities of flavonoids, phenolic acids and saponins in fava beans^30^.

### Functional annotation and enrichment analysis of differential metabolites

**Figure 5:**
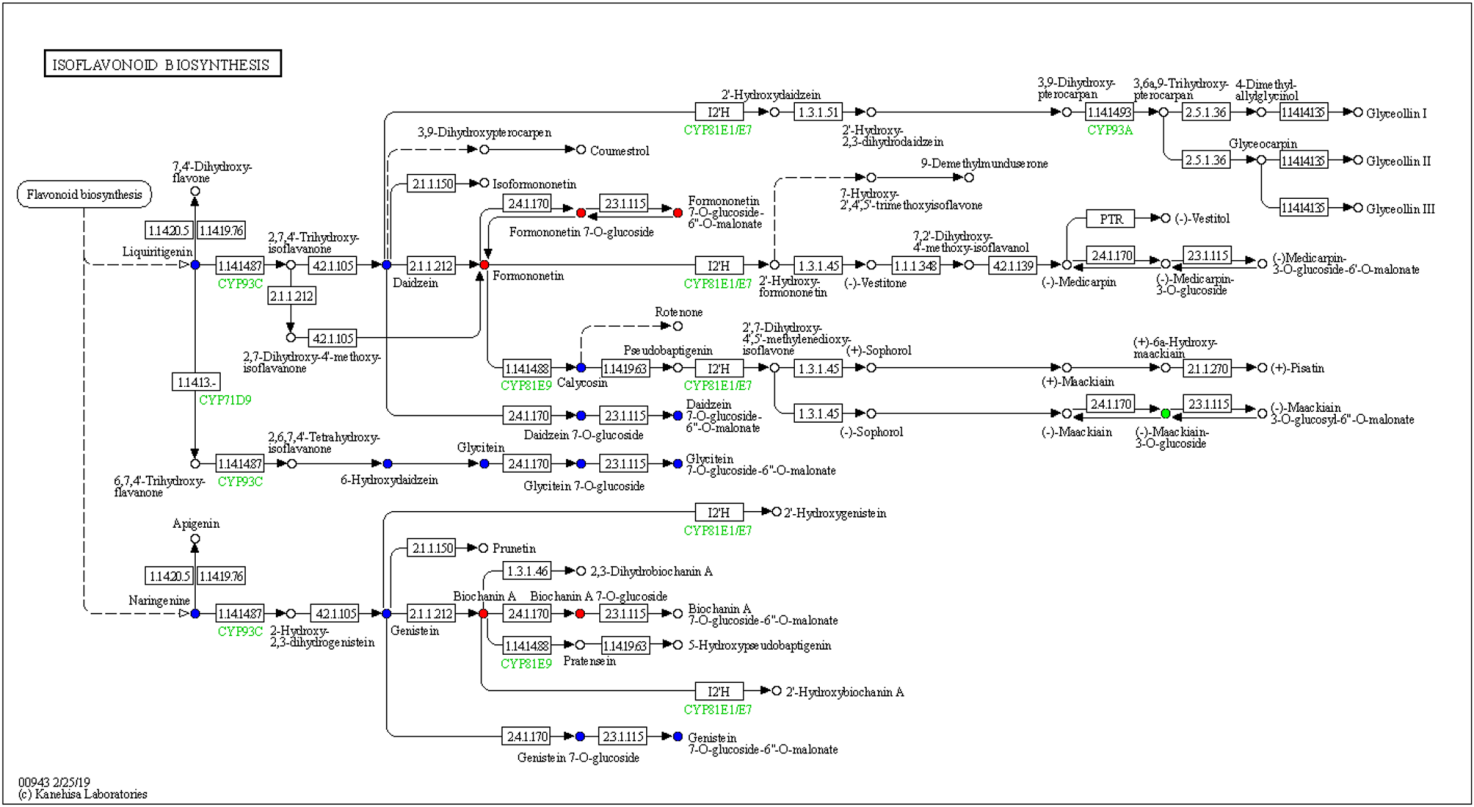
Differential metabolite KEGG pathway diagram. Note: Red indicates that the metabolite content is significantly up-regulated in the experimental group, and blue indicates that the metabolite is detected but not significantly changed. Green indicates that the metabolite content is significantly down-regulated in the experimental group.

In the homepage of the KEGG website, we directly enter the keyword "Flavonoids" and the top 4 pathways are all pathways involving flavonoid metabolites, including:flavonoid synthesis, anthocyanin synthesis, isoflavone synthesis, flavonoids and flavonols synthesis. Using the KEGG database^44^ to annotate and display the differential metabolites identified in these samples, 6 metabolite were significantly up-regulated in the isoflavone synthesis pathway, 1 metabolite was significantly down-regulated, and 13 metabolites did not change significantly (Fig. 5).

**Figure 6:**
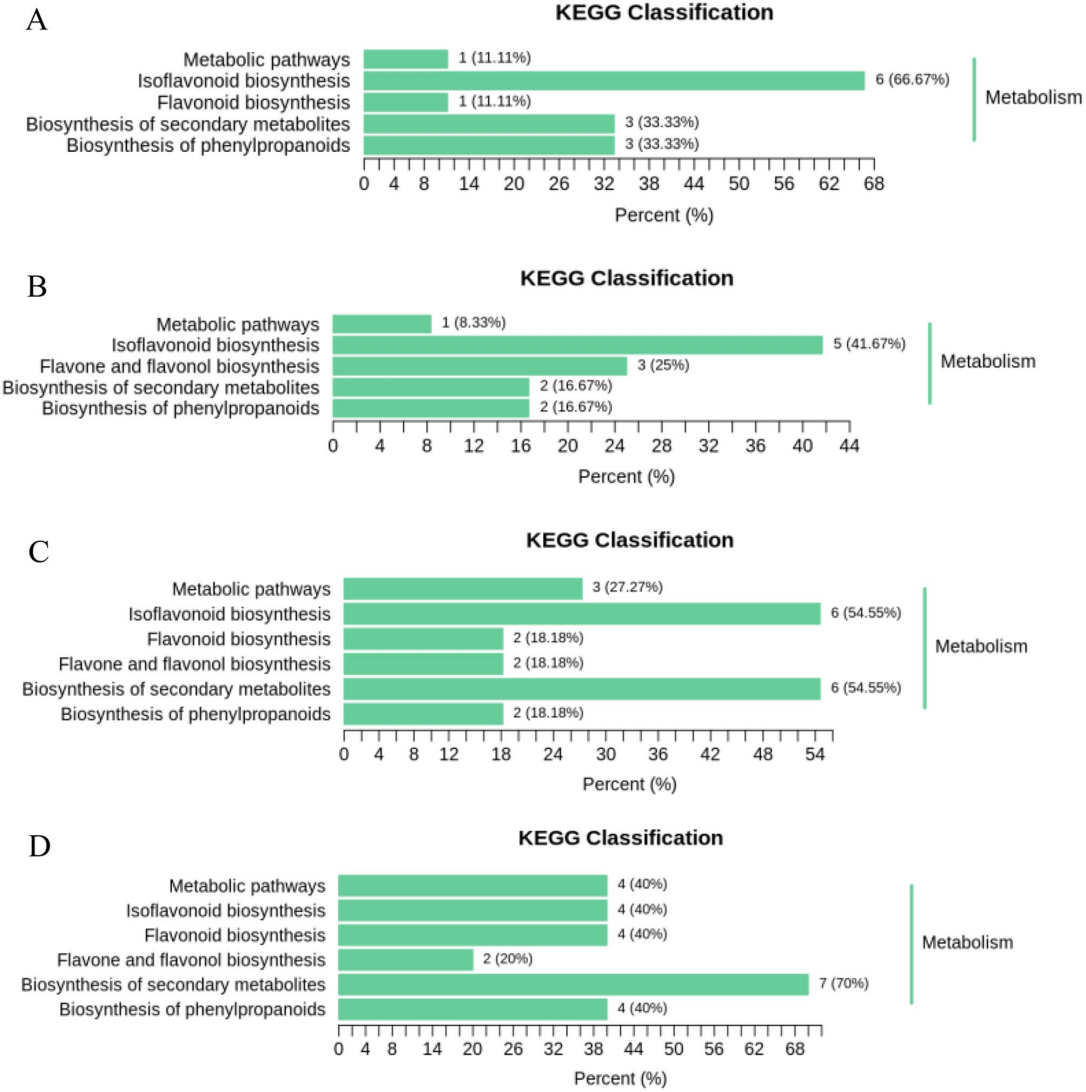
Differential metabolite KEGG classification chart. (**A**): HN52 vs HN71; (**B**): HNS52 vs HNS71; (**C**): HN52 vs HNS52; (**D**): HN71 vs HNS71. Note: The ordinate is the name of the KEGG metabolic pathway, and the abscissa is the number of metabolites annotated to the pathway and its proportion to the total number of metabolites annotated.

There are six primary metabolic pathways for differential metabolism of flavonoids; isoflavonoid biosynthesis, flavonoid biosynthesis, flavone and flavonol biosynthesis, secondary metabolite biosynthesis, and phenylpropanoid biosynthesis (Fig. 6). Unlike soybean seeds, soybean sprouts have flavone and flavonol biosynthesis. Isoflavonoid biosynthesis accounts for most of the pathways, and the proportions in different samples are 66.67% in HN52 vs HN71 (Fig. 6A), 41.67% in HNS52 vs HNS71 (Fig. 6B), 54.55% in HN52 vs HNS52 (Fig. 6C), and 40% in HN71 vs HNS71 (Fig. 6D), respectively. Another important pathway is the biosynthesis of secondary metabolites, and the proportions in different samples are 33.33% in HN52 vs HN71 (Fig. 6A, 16.67% in HNS52 vs HNS71 (Fig. 6B), 54.55% in HN52 vs HNS52 (Fig. 6C), 70% in HN71 vs HNS71 (Fig. 6D), respectively). Metabolic pathway data explained why isoflavones were higher in different metabolites.

**Figure 7:**
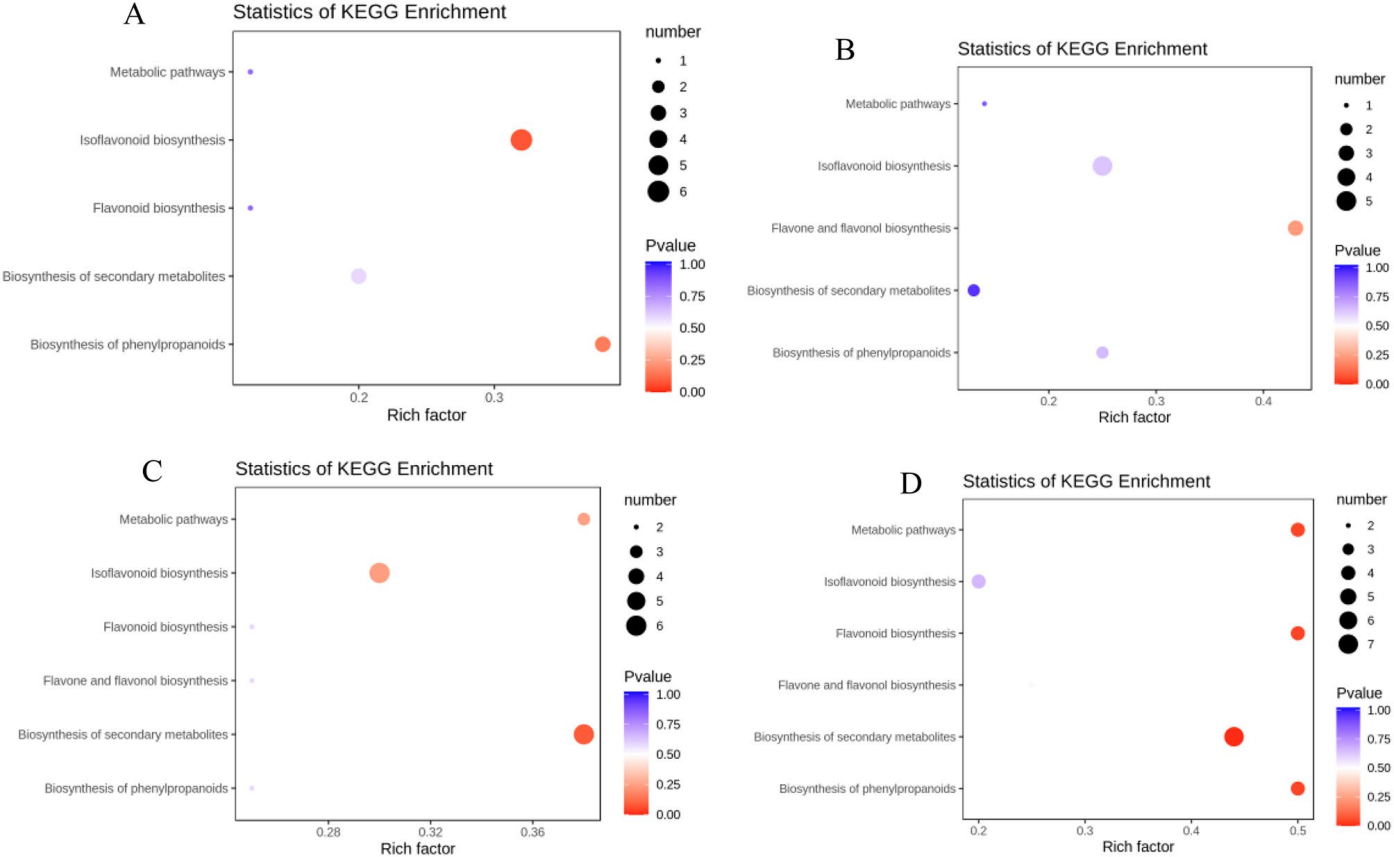
Differential metabolite KEGG enrichment map. (**A**): HN52 vs HN71; (**B**): HNS52 vs HNS71; (**C**): HN52 vs HNS52; (**D**): HN71 vs HNS71. Note: The abscissa represents the rich factor corresponding to each channel, the ordinate is the channel name, and the color of the point is p value. The more red, the more significant the enrichment. The size of the dot represents the number of different metabolites enriched.

As shown in Fig. 7, that the larger size dot indicated isoflavonoid biosynthesis and biosynthesis of secondary metabolites representing the number of these metabolites enriched. The red point represented isoflavonoid biosynthesis in Fig. 7C, indicating that isoflavonoid was more significantly enriched. The redder points indicating isoflavonoid biosynthesis in Fig. 7A, showed that isoflavonoids were more significantly enriched.

## Conclusion

In this study, two soybean varieties with high isoflavone content and their soybean sprouts in Heilongjiang Province, China were analyzed with flavonoid metabolome analysis. 114 differential metabolites were detected which contained 41 isoflavones and 26 flavonoids. These experimental results revealed that the differentially expressed and up-regulated metabolites in soybean sprouts increased compared to soybean seeds. The flavonoid metabolites in the same variety of soybean seeds and sprouts differ greatly in multiples, indicating that flavonoid metabolism is very active during soybean germination, and germination may be a way to accumulate flavonoids. Metabolic pathway data explained why isoflavones were higher than different metabolites. This study detected and analyzed the changes of flavonoid metabolites after soybean germination, providing data reference for the research of flavonoids, and providing ideas for future research on plant secondary metabolism.
